# Assessment of liver fibrosis in children with intestinal failure and prolonged parenteral nutrition

**DOI:** 10.1007/s00383-026-06633-x

**Published:** 2026-09-18

**Authors:** Phoenix WY Wong, Fanny Yeung, Patrick HY Chung, Kenneth KY Wong

**Affiliations:** Division of Paediatric Surgery, Department of Surgery, School of Clinical Medicine, The University of Hong Kong, Hong Kong, Hong Kong S.A.R., China

**Keywords:** Intestinal failure, Transient elastography, Liver fibrosis, IFALD

## Abstract

**Background:**

Children with intestinal failure (IF) often necessitates prolonged parenteral nutrition (PN) use, which predisposes them to IF-associated liver disease (IFALD) and fibrosis. The aim of the study is to evaluate the prevalence and severity of liver fibrosis using transient elastography (TE) on children with IF who were on prolonged PN.

**Methods:**

Patients with IF requiring PN for more than 3 months between Jan 2000 to December 2024 were included in the study. Liver fibrosis was assessed by TE and defined by a fibrosis score (FS) > = 6.8 kPa. Baseline demographics, diagnosis, operation details, PN characteristics, and outcomes were compared between the fibrosis and non-fibrosis groups.

**Results:**

37 children suffered from IF requiring prolonged PN were identified, and TE was performed in 19. 17 patients (89.5%) had massive bowel resection, and 2 (10.5%) had visceral myopathies. Necrotizing enterocolitis was the commonest cause of IF in this cohort (14, 73.7%). 8 (42.1%) remained dependent on PN at the time of TE. Median PN duration was 16 months (3–144 months). 4 patients (21.1%) had liver fibrosis on TE. The fibrosis and non-fibrosis groups (15, 78.9%) were similar in terms of baseline demographics, operative details and PN characteristics. There were 2 deaths in the fibrosis group, one from IF related liver failure and the other from respiratory failure with known cholestasis. (50% vs. 0%, *p* = 0.035).

**Conclusion:**

With TE measurement, the incidence of liver fibrosis in this cohort was at 21%, which appears to be higher than previously reported. They are associated with higher risk of mortality but not with known risk factors of IFALD. Further prospective studies with larger sample size will be required for proper evaluation.

## Introduction

Intestinal failure (IF) in children could result from various congenital or acquired conditions leading to anatomical or functional loss of bowel mass. Prolonged parenteral nutrition (PN) is vital in order to sustain life and promote growth in children with IF, while it can also predispose them to a number of IF-related complications and mortality. With the introduction of intestinal rehabilitation program and bowel lengthening surgeries, the outcomes of children in IF are gradually improving with 47–70% of them being able to achieve enteral autonomy in the long run [1, 2].

Among known IF-related complications, IF-associated liver disease (IFALD), with a reported prevalence at 20–25% [3], remains a significant cause of morbidity and death in children with IF with prolonged PN use. It is defined as hepatobiliary dysfunction arising as a consequence of medical and surgical management strategies for IF, typically characterized by cholestasis [3]. The development of IFALD is likely multifactorial, and current mainstream of monitoring relies on serial biochemical testing and/or liver biopsies. Emerging evidences demonstrated that liver injury/fibrosis resulted from IFALD could persist despite biochemical resolution of cholestasis and normalization of other previously deranged parameters, namely albumin and prothrombin time [4, 5]. Repeated liver biopsies for the purpose of monitoring of IFALD is also not favoured, as it is an invasive procedure with 1–5% complication rate, carries risk of sampling error, inter and intra-observer variation, and the need of general anaesthesia in children [6]. There is a lack of reliable and reproducible tool for monitoring of IFALD and evaluation of long term sequalae on liver fibrosis after prolonged PN in children.

Liver transient elastography (TE) using Fibroscan^®^ (Echosens, France) has emerged in recent years as a non-invasive tool for evaluation of liver fibrosis. The degree of liver stiffness is measured by the velocity of shear wave induced to the liver via a mechanical probe, which in turn reflects the amount of fibrotic tissue in liver. Liver fibrosis score (FS) is then expressed in kiloPascal (kPa).

The primary objective of the study was to evaluate the presence and severity of liver fibrosis in IF children with a history of long term PN using TE. Those with and without liver fibrosis on TE were compared to identify potential risk factors of IFALD development. The secondary aim was to prospectively correlate FS to the biochemical changes of liver fibrosis and clinical development of portal hypertensive complications.

## Methods

### Study design

This was a prospective single-centre observational study. All patients younger than 18 years old who required prolonged PN (defined by a minimum of 90 days on consecutive PN use [7, 8]) for IF from January 1997 to December 2024 were eligible for the study. Those with concomitant liver disease that is not related to PN use or liver impairment secondary to cardiac disease were excluded from the study.

After enrolment, patients underwent TE using Fibroscan^®^ performed by two experienced operators. For TE, 10 valid measurements were taken and FS was calculated from their median. FS was only considered valid per instructions of the manufacturer: if the success rate of obtaining valid measurements was greater than 60% and the ratio of interquartile range/median (IQR/M) was less than 30% [9]. On a more technical aspect, 2 probes were commercially available for TE measurement: the M probe and the S2 probe. In compliance with the manufacturer’s recommendations, the S2 probe was used for TE measurement in patients younger than 12 years old, while those older would have measurement done by the M probe. Liver fibrosis, in the context of this study, was defined by having a FS greater than 6.8 kPa based on published literatures [10, 11].

Data on baseline demographics, operative details, nutrition characteristics, biochemical/radiological/endoscopic investigations, and outcomes were collected. Investigation results relevant to liver function status and portal hypertension presence were also included for analysis, namely: (1) biochemical parameters of liver function including serum bilirubin and transaminases levels, platelet counts and prothrombin level; (2) radiological, endoscopic and/or clinical findings of portal hypertensive complications. Liver biopsy result was also included for analysis if available. The definition of cholestasis, portal hypertension, catheter related blood stream infection (CRBSI) and small bowel bacterial overgrowth (SBBO) followed the ESPGHAN and NASPGHAN guideline [12, 13].

### Setting

Our centre provides tertiary paediatric surgical services covering a network of hospitals. Patients with IF were routinely reviewed by our unit and the paediatricians to provide a holistic care in ensuring adequate nutrition for growth and development as well as monitoring of potential complications. Regular blood-taking and/or imaging were arranged for patients who remained dependent on PN. For those who achieved enteral autonomy successfully, regular outpatient reviews would be continued and investigations may be arranged if necessary.

### Statistical analysis

Statistical analysis was performed with SPSS Statistics Version 26 (SPSS Inc., Chicago, IL., USA). Continuous and categorical variables were expressed as medians with range and frequency with percentage respectively. The Mann-Whitney U test was used for comparison of continuous variables, and Pearson’s chi-squared test for comparing categorical variables. Correlations with FS were calculated using Point-Biserial correlation for binary variable and Kendall Tau’s correlation for continuous variable given the small sample size of the study. A strong correlation was implied by a coefficienct greater than 0.5, while a coefficient between 0.3 and 0.5 suggested a moderate correlation. Statistical significance was denoted by a p-value less than 0.05.

The study was reviewed and approved by the hospital’s institutional review board (reference no. UW 25–473).

## Results

37 children required prolonged PN due to IF were identified. 19 were recruited into the study (8 males and 11 females) and the majority of them were born preterm (73.7%). Comorbidities were present in 5 patients (26.3%). Regarding the cause of intestinal failure, necrotizing enterocolitis (NEC) was the most common (73.7%), followed by malrotation volvulus and visceral myopathies. All patients in this cohort underwent operation at median age of 2 weeks old, and most underwent bowel resections (89.5%) with median length of remaining small bowel at 22 cm (Table 1) 15 (78.9%) had stoma created during the indexed operation, in which 10 were subsequently closed before TE measurement. Portal hypertensive complications occurred in 2 patients, namely splenomegaly (2, 10.6%), variceal bleeding (1, 5.3%) and hepatic encephalopathy (2, 10.6%).

**Table 1:** Patients' characteristics, median (range) or number (percentage)

Demographical information (n=19)	
Male: female	8:11
Gestation at birth, weeks	30 (27–39)
Term at birth	5 (26.3%)
Birth weight, kg	1.43 (0.92–4.2)
Primary disease	
NEC	14 (73.7%)
Malrotation volvulus	2 (10.5%)
Meconium peritonitis	1 (5.3%)
Hirschsprung disease/ visceral myopathy	2 (10.6%)
Comorbidity	5 (26.3%)
Congenital heart disease	1
Solitary kidney	1
Primary immunodeficiency	1
Encephalomalacia	1
Austic spectrum disorder	1
Operative details	
Age at first operation, weeks	2 (0–52)
Gestation at first operation, weeks	32 (29–90)
Bowel resection	17 (89.5%)
Length of remaining small bowel, cm	22 (4–200)
Ileocaecal valve resection	10 (52.6%)
Colon resection	10 (52.6%)
Stoma creation	15 (78.9%)
Stoma present at FS	5 (26.3%)
Bowel lengthening procedure	1 (5.3%)
Nutritional characteristics	
Duration of PN, months	16 (3–144)
Enteral feeding during PN use	18 (94.7%)
Enteral autonomy	11 (57.9%)
Lipid regime in PN	
SMOF (soy, MCT, omega-3, fishoil)	10 (52.6%)
Omegaven	11 (57.9%)
Omega-3 content	15 (78.9%)
Low lipid regime	6 (31.6%)
Lipid modification	4 (21.1%)
IFALD disease profile	
History of cholestasis when on PN	17 (89.5%)
Persistent cholestasis at TE	5 (26.3%)
History of CRBSI	17 (89.5%)
History of SBBO	11 (57.9%)
Portal hypertensive complications	2 (10.6%)
Ursodeoxycholic acid use	14 (73.7%)
Oral antibiotics prophylaxis	9 (47.4%)
Liver biopsy performed	3 (15.8%)
Outcomes	
Mortality	2 (10.6%)

*NEC* necrotizing enterocolitis, *PN* parenteral nutrition, *CRBSI* catheter related blood stream infection

The patients were on PN at a median of 11.5 months (3–144 months). Enteral autonomy was achieved in 11 (57.9%) prior to TE measurement. Majority of patients had omega-3 fatty acid in PN in the form of SMOFlipid→ or omegavenⓇ (Fresenius Kabi, Germany). 17 patients (89.5%) had a history of PN-related cholestasis, most cholestasis resolved after modification of PN regime, with only 5 (26.3%) had persistent cholestasis at the time of TE.

Regarding the risk factors for development of IFALD, majority of the patients in this cohort had history of CRBSI and SBBO, with around half had previously been on oral antibiotics prophylaxis for gut decontamination. There were 2 deaths in this cohort (10.6%) at conclusion of study period: 1 from liver failure due to IFALD at 11 months old with the highest FS of 26 kPa, and 1 from pneumonia with background PN-related cholestasis at 5 years old(FS of 13.5 kPa). Both had history of portal hypertensive complications prior to their demise.

TE was performed at a mean age of 8 years old (0–17 years old), patients’ PN use ranging from still dependent to having been discontinued for 191 months. The median FS was 5.65 kPa (3.4–26 kPa) (Table 2).

**Table 2:** TE measurement details, median (range) or number (percentage)

Age at TE, years	8 (0–17)
Duration between PN discontinuation and TE measurement, months	59 (0–191)
FS, kPa	5.8 (3.3–26)
FS ≥ 6.8 kPa	4 (21.1%)

*TE* transient elastography, *PN* parenteral nutrition, *FS* fibrosis score

4 patients (21.1%) had persistent liver fibrosis on TE. (Table 3) All of them suffered from NEC with major bowel resection performed, with a median 14.5 cm remaining small bowel length. 2 out of 4 patients in the fibrosis group had significant comorbidities, namely encephalomalacia and Job syndrome respectively. In the fibrosis group, 3 had concomitant deranged biochemical liver parameters with either hyperbilirubinemia or elevated alkaline phosphatases, with liver biopsies performed confirming presence of bridging fibrosis histologically. All 3 of them remained dependent on PN at the time of TE measurement. As mentioned ealier, 2 in the fibrosis group who suffered from portal hypertensive complications had passed away. One patient remained TPN dependent with no portal hypertensive complications on radiological and endoscopic screening. The biochemical parameters of liver function remained slightly deranged namely the bilirubin and the transaminases level. The other patient, aged 11 at the time of TE, had achieved enteral autonomy for more than 10 years and had resolution of cholestasis biochemically. The family has declined liver biopsy and endoscopic screening for varices after TE measurement due to fear of invasive nature of the tests and need of anesthesia, hence the validity of FS cannot be be further verified for this patient.

When comparing the fibrosis and non-fibrosis group, patients in the fibrosis group underwent TE at a younger age. Larger portion of patient in the fibrosis group remained dependent on PN and all deaths were found in the fibrosis group. The two groups did not differ significantly as shown in Table 3, in particular on known risk factors (namely prematurity, short bowel length, history/number of SBBO/CRBSI, high lipid profile in PN) and possible protective factors (use of omega-3 based lipid, lipid modification in PN, ursodeoxycholic acid and oral antibiotics prophylaxis) of IFALD (Table 3).

**Table 3:** Comparison between significant fibrosis group and non-fibrosis group on transient elastography, median (range) or number (percentage)

	Fibrosis group (n = 4)	Non-fibrosis group (n = 15)	*P *value
Baseline characteristics			
Male: female	2:2	6:9	1
Gestation at birth, weeks	31.5 (27–39)	29.5 (27–38)	0.42
Term at birth	1 (25%)	4 (26.7%)	1
Birth weight, kg	1.58 (1–2.74)	1.43 (0.92–4.2)	1
Primary disease			0.239
NEC	4 (100%)	10 (66.7%)	
Malrotation volvulus	0	2 (13.3%)	
Meconium peritonitis	0	1 (6.7%)	
Hirschsprung disease/ visceral myopathy	0	2 (13.3%)	
Comorbidity	2 (50%)	1 (6.7%)	0.097
Operative information			
Age at operation, weeks	1.5 (0–4)	2 (0–52)	0.645
Gestation at operation, weeks	34 (29–39)	32 (29–90)	0.42
Length of remaining small bowel, cm	14.5 (4–45)	28 (9.5–200)	0.109
Ileocaecal valve resection	3 (75%)	7 (46.7%)	0.582
Colon resection	3 (75%)	7 (46.7%)	0.582
Stoma creation	4 (100%)	11 (73.3%)	0.53
Bowel lengthening surgery	0	1 (6.7%)	1
Nutritional characteristics			
Duration of PN, months	12.5 (3–16)	40 (3–144)	0.262
Enteral autonomy	1 (25%)	10 (66.7%)	0.214
Lipid regime in PN			
SMOF	3 (75%)	7 (46.7%)	0.582
Omegaven	2 (50%)	9 (60%)	1
Omega-3 content	3 (75%)	12 (80%)	1
Low lipid regime	2 (50%)	4 (26.7%)	0.557
Lipid modification	2 (50%)	4 (26.7%)	0.557
IFALD disease profile			
PN-related cholestasis	4 (100%)	13 (86.7%)	1
History CRBSI No. of CRBSI SBBO No. of SBBO	4 (100%) 2.5 (2–3) 2 (50%) 1.5 (0–3)	13 (86.7%) 3 (0–6) 9 (60%) 1 (0–4)	1 0.749 1 0.752
Portal hypertensive complications	2 (50%)	0	0.035
Ursodeoxycholic acid	4 (100%)	10 (66.7%)	0.53
Oral antibiotics decontamination	1 (25%)	8 (53.3%)	0.582
TE details			
Age at TE, years	2.5 (0–11)	8 (5–17)	0.056
Duration between PN discontinuation and TE measurement, months	0 (0–131)	74 (0–191)	0.275
Outcome			
Mortality	2 (50%)	0	0.035

*CRBSI* catheter related blood stream infection, *SBBO* small bowel bacterial overgrowth, *FS* fibrosis score, *NEC* necrotizing enterocolitis, *PN* parenteral nutrition, *TE* transient elastography

A higher fibrosis score was correlated with occurrence of portal hypertensive complications (correlation coefficient 0.885, *p* < 0.001), while there was moderate positive correlation with serum bilirubin (correlation coefficient 0.433, *p* = 0.013) and INR level (correlation coefficient 0.333, *p* = 0.07) (Table 4).

## Discussion

Prolonged PN is required for children with IF to sustain life, promote growth and development before intestinal adaptation, but it could also lead to significant morbidity and mortality. While early stages of IFALD is reversible, late detection and treatment could lead to progression into liver failure and portal hypertensive complications which could be potentially life-threatening. Advances in intestinal rehabilitation over the last two decades, with joint medical and surgical care such as modified lipid emulsions and bowel lengthening surgery, have led to improvement in outcomes in children suffering from IF. Yet, the incidence of IFALD is still reported to be as high as 20–25% [3].

The exact aetiology of IFALD is uncertain, but is likely multifactorial with interplay from both patients’ characteristics and treatment modality for IF. Immature liver in younger children, prolonged PN use, lack of enteral stimulation and recurrent sepsis were factors related to progression of IFALD.[3] The disease commonly presents with persistently elevated serum bilirubin level and/or other transaminases, and can be confirmed with a liver biopsy. Although PN-related cholestasis may resolve biochemically after cessation of PN, current evidences have demonstrated that liver fibrosis and/or cirrhosis can persist and even progress. [4, 14] Early recognition and continuous monitoring of established liver damage in this condition is vital before hepatic decompensation, as end-stage liver disease can be found in up to 4% patients with IFALD [3].

TE with Fibroscan^®^ has shown promise as a non-invasive modality in assessing the degree of liver fibrosis. Comparing with liver biopsy, which is the gold standard in the diagnosis of liver fibrosis, it can be safely performed at bedside and carries the advantages of being non-invasive and avoiding the risks of complications, anesthesia and sampling error. Serial measurements can also be performed with ease for long term monitoring of liver fibrosis degree. A FS of 6.8 kPa was chosen as the cut-off of upper limit in this cohort. This is derived from our centre’s experience in performing TE for 37 children with biliary atresia post-Kasai operation, in which a FS greater than 6.8 kPa was significant associated with higher aspartate transaminase platelet ratio index (APRI) and FIB-4 score as well as larger splenic diameter [15]. This value is also similar to previously reported cut off references in large cohort of healthy paediatric population [10, 11]. However we recognize that the use of TE has not been reported in larger cohort of children with IF previously, and the cut-off of 6.8 kPa may not translate well into this population specifically. Prospective study pairing TE measurement and liver histology will be required to provide further information on the validity of this cut-off value in children with IF. As this study was performed within a relatively short window on mostly younger patients, long-term study is also warranted to delineate the outcomes in the long run e.g. portal hypertensive complications, liver failure rate and need of liver transplantation etc.

**Table 4:** Correlation analysis with TE

	Correlation coefficient	*P* value
Platelet	0.007	0.970
Serum bilirubin	0.433	0.013
INR	0.333	0.07
Prothrombin time	0.282	0.183
APRI	0.292	0.297
FIB4	0.097	0.572
Portal hypertensive complications	0.885	<0.001

*TE* transient elastography, *APRI* aspartate transaminase platelet ratio index

The prevalence of liver fibrosis based on TE was 21.1% in this cohort of children, which was comparable to previously published figures [3, 7]. The majority of those with liver fibrosis remained PN-dependent, which is a known risk factor of IFALD. Of note, 1 patient who had been taken off PN for more than a decade was found to have liver fibrosis on TE. Although sampling error is a possibility, this finding also supported that IFALD and its liver damage can persist or may even progress after weaning off PN and biochemical resolution. This highlights the necessity in continuous follow-up and monitoring of liver function in patients with IF and history of prolonged PN, and they warrant proper screening programme for portal hypertensive complications.

As clinicians taking care of the paediatric population, it is not uncommon that patients and/or family experienced significant anxiety with repeated blood taking or invasive procedures such as endoscopy and/or liver biopsy. As demonstrated in this study, FS has a positive correlation with the occurrence of portal hypertensive complications, serum bilirubin as well as the INR level. It also echoes the finding in adult counterpart, in which there is a significant correlation between FS and serum bilirubin level [16]. While the correlation of FS and biochemical parameters of liver function has not been validated in large scale study in children, our finding raises the possibility of using TE as a continuous and serial monitoring modality in addition to blood taking, as its non-invasive nature can help lower patient’s and parenteral anxiety. With serial measurements, an increasing FS on TE can also encourage patients to participate in the screening programme of liver function and portal hypertensive complications.

Two deaths were included for analysis in this cohort at the time of reporting in the fibrosis group, and both occurred at a relatively young age. Although this finding is statistically significant, the causes of all deaths were not all directly attributable to IFALD. The patients in the fibrosis were relatively younger and had more associated significant comorbidity, while there was no significant differences in terms of known risk factors of IFALD aforementioned between the fibrosis and non-fibrosis groups [7, 17]. Apart from potential selection bias and limitation of the sample size, it is possible that there are other components at play e.g. genetics, which predispose certain patients at risk of associated anomalies and a more rapid deterioration in terms of liver impairment and clinical course.

There were a number of limiting factors in the study. Firstly, the small sample size has reduced the availability of the study and selection bias is inevitable being a single centre observational study. In addition, liver biopsies were not performed for all patients for proper correlation between FS and liver fibrosis. FS can also be affected by multiple factors, including the presence of cholestasis, nutritional status, movement artefact etc [10]. While it will be ideal to pair FS result with liver biopsies, patients and/or their families often found the procedure daunting due to the invasive nature and the need of anesthesia. With our results, there will be more evidence to encourage patients with a high FS to undergo liver biopsy for diagnosis while also carrying prognostic significance.

## Conclusion

With TE measurement, the incidence of liver fibrosis in this cohort was at 21%, which appears to be higher than previously reported. They are associated with higher risk of mortality but not with known risk factors of IFALD. Hence early identification and close monitoring of at-risk patients are warranted. Further prospective longitudinal studies with larger sample size will be required for proper evaluation of validity of TE in children with IF and its association with the biochemical parameters of liver function. TE may be useful as an adjunct in long-term management of children with IF.

## Data Availability

No datasets were generated or analysed during the current study.
